# Association of Sleep Patterns and Lifestyles With Incident Hypertension: Evidence From a Large Population-Based Cohort Study

**DOI:** 10.3389/fcvm.2022.847452

**Published:** 2022-04-01

**Authors:** Yanling Lv, Guanhua Jiang, Xiao Tan, Wei Bao, Liangkai Chen, Liegang Liu

**Affiliations:** ^1^Hubei Key Laboratory of Food Nutrition and Safety, Department of Nutrition and Food Hygiene, School of Public Health, Tongji Medical College, Huazhong University of Science and Technology, Wuhan, China; ^2^Ministry of Education Key Lab of Environment and Health, School of Public Health, Tongji Medical College, Huazhong University of Science and Technology, Wuhan, China; ^3^Department of Neuroscience, Uppsala University, Uppsala, Sweden; ^4^Department of Clinical Neuroscience, Karolinska Institutet, Stockholm, Sweden; ^5^Department of Epidemiology, College of Public Health, University of Iowa, Iowa City, IA, United States

**Keywords:** sleep pattern, lifestyle, hypertension, prospective cohort study, UK Biobank

## Abstract

**Background:**

Adherence to a healthy lifestyle (no smoking, consuming a healthy diet, engaging in physical activity, and maintaining a healthy weight) is recommended in current guidelines for hypertension prevention. However, evidence regarding the association between sleep behaviors, independently and jointly with traditional lifestyle factors, and the risk of hypertension is limited.

**Methods:**

This prospective study included 165,493 participants who are free of hypertension at baseline from the UK Biobank. Sleep behaviors, including chronotype, sleep duration, insomnia, snoring, and daytime sleepiness were used to construct a healthy sleep score. We also derived a healthy lifestyle score based on smoking status, diet quality, physical activity, and body mass index (BMI). Cox proportional hazards regression models and competing risk analyses were used to estimate the associations of the healthy sleep score and healthy lifestyle score with the risk of hypertension. The population attributable risk percent (PAR%) was estimated for increased cases of hypertension due to poor adherence to a healthy sleep pattern or a healthy lifestyle.

**Results:**

A total of 10,941 incident hypertension cases were documented during a median of 11.8 years of follow-up. The multivariable-adjusted hazard ratio (HR) for hypertension was 0.58 [95% confidence interval (CI): 0.52, 0.65] for participants with a sleep score of 5 compared with 0–1, and 0.48 (95% CI: 0.43, 0.54) for participants with a lifestyle score of 4 compared with those who scored 0. For joint association, those with a poor sleep pattern and a poor lifestyle had the highest risk of hypertension [HR: 2.41 (95% CI: 2.12, 2.74)]. PAR% was 14.7% (95% CI: 12.3%, 17.1%), 20.1% (95% CI: 17.6%, 22.6%), and 31.7% (95% CI: 27.6%, 35.6%) for poor adherence to a healthy sleep pattern, a healthy lifestyle, and the combination of a healthy sleep pattern and a healthy lifestyle.

**Conclusion:**

Both a healthy sleep pattern and a healthy lifestyle were associated with a lower risk of hypertension, and the benefits of adhering to a healthy sleep pattern complement the well-established lifestyle for the optimal primary prevention of hypertension. These findings support the current perspective that a healthy sleep pattern is an important part of a healthful and productive lifestyle for hypertension prevention.

## Background

Hypertension is the leading risk factor for cardiovascular morbidity and mortality and accounted for 10.8 million (19.2% of all deaths) attributable deaths in 2019 globally (1, 2). Previous studies (3–6) and current guidelines (7, 8) advocate lifestyle, including smoking, diet, physical activity, and BMI, as one of the important modifiable risk factors for hypertension, and the association of the overall lifestyle with incident hypertension has been shown in several studies (9–11). Aside from these broadly recommended lifestyle factors, increasing evidence has implicated that sleep behaviors, such as chronotype, sleep duration, insomnia, snoring, and daytime sleepiness, are related to hypertension events (12–15). However, sleep behaviors typically interplay with each other, whereas most previous studies focus on individual behavior without considering the combined effect of other behaviors. Only few studies have taken several sleep behaviors together and assessed their joint association with hypertension risk. Limited evidence suggests that insomnia with short sleep duration is associated with the highest risk of hypertension among the sleep behaviors (16). Meanwhile, no prospective study, to our knowledge, has assessed the association of all the five sleep-related factors (i.e., chronotype, sleep duration, insomnia, snoring, and daytime sleepiness) with the risk of hypertension. Moreover, the interaction between sleep behaviors and lifestyles, as well as the joint association of sleep behaviors and lifestyles with hypertension, is still unclear.

Therefore, we aimed to estimate the association of the combination of the five sleep behaviors and overall lifestyle with the risk of hypertension independently and jointly. Meanwhile, we calculated the population attributable risk percent (PAR%) to estimate the percentage of hypertension events that theoretically will not occur if all participants had a healthy sleep pattern, a healthy lifestyle, or both at the same time.

## Materials and Methods

### Study Design and Participants

The study design and population of the UK Biobank have been reported in detail previously (17). Briefly, the UK Biobank is a large-scale prospective cohort with more than 500,000 participants aged 37–73 years who attended 1 of 22 assessment centers across the United Kingdom between 2006 and 2010.^1^ At baseline recruitment, participants completed touch-screen questionnaires and a standardized interview, had physical measurements taken, and provided biological samples. Data from the health care system were also collected. The UK Biobank study was approved by the North West Multi-centre Research Ethics Committee (REC reference for UK Biobank 11/NW/0382), and all participants provided written informed consent.

In the present analysis, we excluded participants who withdrew from the UK Biobank (*n* = 31), those who were pregnant or unsure of their pregnancy status at baseline (*n* = 371), those with hypertension at baseline (*n* = 280,273), and those with missing value on variables used to define a healthy sleep pattern (*n* = 39,291) and a healthy lifestyle (*n* = 17,046), finally, 165,493 participants were included in this analysis (Supplementary Figure 1).

### Assessment of Incident Hypertension

At baseline, blood pressure was measured twice a few minutes apart using Omron 705 IT electronic blood pressure monitor. We calculated the mean systolic blood pressure (SBP) and diastolic blood pressure (DBP) from two automated BP readings (*n* = 456,936). For participants with at least one missing automated BP reading, the mean of two manual BP measurements (*n* = 28,699), or a single automated BP reading (*n* = 15,421), or a single manual BP measurement (*n* = 15,099) was used instead. A threshold of ≥ 140/90 mm Hg was used to determine baseline hypertension according to the 2018 European Society of Cardiology (ESC)/European Society of Hypertension (ESH) Guidelines for the management of arterial hypertension (7). Recorded hypertension events before recruitment, self-reported use of BP-lowering medication, and doctor-diagnosed hypertension at recruitment were considered baseline hypertension. Participants with hypertension at baseline were all excluded from our analysis.

Incident hypertension was ascertained based on hospital admission and diagnosis data and death registry records linked to the UK Biobank according to ICD-9 and ICD-10 (the 9th and 10th revisions of the International Classification of Diseases, *n* = 10,057). The ICD code and UK Biobank Field ID of hypertension are shown in Supplementary Table 1. The recorded date of hypertension diagnosis was used as the time of the event. The admission date was used as a substitute for participants with a missing diagnosis date. Self-reported physician-diagnosed hypertension and use medication for lowering blood pressure during follow-up were also used to double check the disease ascertainment (*n* = 884). Self-reported age of hypertension diagnosis was used to help calculate the period of the disease.

### Assessment of Healthy Sleep Behaviors

All sleep behaviors were self-reported and recorded using an electronic questionnaire. Questions about sleep behaviors have been described in detail (18). Five sleep factors (chronotype, duration, insomnia, snoring, and excessive daytime sleepiness) were used to generate a healthy sleep score and low-risk sleep behaviors were defined as follow (18): early chronotype (“morning” or “more morning than evening”), ideal sleep duration (7–8 h/day), reported never/rarely insomnia, no snoring, and reported no excessive daytime sleepiness (“never/rarely” or “sometimes,” Supplementary Table 2). We scored each sleep behavior with 1 point and summed the five sleep factors score to constitute a healthy sleep score ranging from 0 to 5, where a higher score indicating a healthier sleep pattern. We categorized sleep patterns into healthy (4 ≤ healthy sleep score ≤ 5), intermediate (2 ≤ healthy sleep score ≤ 3), and poor (0 ≤ healthy sleep score ≤ 1). We also calculated a weighted sleep score based on β coefficients of each sleep factor in the Cox proportional hazard regression model, which included all five sleep factors and adjusted for age, sex, ethnicity, education, household income, Townsend deprivation index, healthy lifestyle categories, baseline mean arterial pressure, alcohol consumption, family history of hypertension, self-reported cardiovascular diseases, diabetes, chronic kidney disease, and cancer. The equation is as follow: weighted score = [(β1 × factor1) + (β2 × factor2) + (β3 × factor3) + (β4 × factor4) + (β5 × factor5)] × (5/sum of the β coefficients). The weighted sleep score also ranged from 0 to 5 with the consideration of the effect of each individual component on the risk of hypertension, and the weighted score was categorized as healthy, intermediate, and poor according to the distribution of the unweighted sleep score (19).

### Assessment of Healthy Lifestyle

A healthy lifestyle score was constructed based on four modifiable lifestyle factors [smoking status, diet, physical activity, and BMI (1)]. Low-risk lifestyle factors were defined according to the American Heart Association guidelines: no current smoking, a healthy diet, regular physical activity, and 18.5 ≤ BMI < 25 kg/m^2^ (Supplementary Table 3). In this analysis, regular physical activity was defined as at least 150 min of moderate activity, 75 min of vigorous activity weekly, or 150 min of moderate and vigorous activities every week (20). We defined a healthy diet according to a previous UK study (21), which considered the increased consumption of fruits, vegetables, whole grains, vegetable oil, fish, and dairy and the decreased consumption of refined grains, unprocessed meats, processed meats, and sugar-sweetened beverages (SSB) based on dietary priorities for cardiometabolic health (22), and we modified it by substituting SSB with Kawasaki formula-estimated daily sodium intake (23), considering the essential role of sodium in the development of hypertension, as well as the unpractical evaluation of SSB intake in the UK Biobank. We defined a healthy diet as adherence to at least four of the items mentioned above. The definitions and variables used for diet components are shown in Supplementary Table 4. For each lifestyle factors, participants received 1 point if he or she met the criterion for the low-risk category or 0 point otherwise. The overall healthy lifestyle score ranges from 0 to 4, and higher scores indicate higher adherence to a healthy lifestyle. We also categorized lifestyles into healthy (3 ≤ healthy lifestyle score ≤ 4), intermediate (healthy lifestyle score = 2), and poor (healthy lifestyle score ≤ 1).

### Statistical Analyses

Cox proportional hazards regression models were used to estimate hazards ratio (HR) and 95% confidence interval (CI) with the years of follow-up as the time metric, and competing risk analyses with cause-specific hazard function were conducted. The time of events was calculated from the baseline recruitment date to the date of the first diagnosis of hypertension, death, lost of follow-up, or censoring date (31 December, 2020), whichever came first. We first investigated the association of healthy sleep scores and healthy lifestyle scores with the risk of hypertension separately, and then we examined the joint association and interaction between sleep pattern categories and lifestyle categories on hypertension events.

Three stepwise models were established to adjust for known or suspected risk factors for hypertension. Model 1 was adjusted for age (continuous) and sex (male or female). Model 2 was adjusted for Model 1 covariates and ethnicity (White, mixed, Asian, Black, Chinese, or others), education (college or university, vocational, upper secondary, lower secondary, or others), Townsend deprivation index quintiles, household income (< £18,000, £18,000–£30,999, £31,000–£51,999, £52,000–£100,000, or > £100,000), baseline mean arterial pressure [MAP, calculated as MAP = (SBP + 2 × DBP)/3], moderate alcohol consumption [0–14 g/day for women and 0–28 g/day for men (22)], and lifestyle categories (for sleep pattern analysis) or sleep pattern categories (for lifestyle analysis). Model 3 was adjusted for Model 2 covariates plus a family history of hypertension (yes or no), baseline cardiovascular disease (CVD, yes or no), diabetes (yes or no), chronic kidney disease (CKD, yes or no), and cancer (yes or no). The definitions of these diseases are shown in Supplementary Table 1. All individual sleep factors or lifestyle factors were included in the final model simultaneously and mutually adjusted to estimate their effects on hypertension risk (Model 4 in Supplementary Tables, if any). Linear trend tests were conducted by treating the healthy sleep score and healthy lifestyle score as continuous variables, and the HRs were interpreted as the hypertension risks associated with per lifestyle score or sleep score increment. The *P*-value for interaction was obtained by the joint test to estimate the statistical significance of the difference between subgroups (24). We calculated PAR% using the fully adjusted Model 3 to estimate the percentage of hypertension events in the study population that theoretically will not occur if all participants had a healthy sleep pattern, a healthy lifestyle, or both (25). We conducted PAR analysis for the sleep and lifestyle scores separately or in combination with healthy lifestyle or healthy sleep pattern to estimate the incremental benefits of sleep and lifestyle factors.

Subgroup analyses and several sensitivity analyses were conducted. We performed stratified analyses to test the association of healthy sleep score with incident hypertension across age, sex, baseline CVD, CKD, diabetes, cancer, and each component of the healthy lifestyle. For sensitivity analyses, we first analyzed the association of healthy sleep scores and healthy lifestyle scores with the risk of hypertension among participants without CVD at baseline recruitment. Second, we excluded those who developed hypertension or died within 2 years from baseline and re-ran the analyses to minimize the reverse causation. Third, we further adjusted for lipid-lowering medication use and diabetes medication use in the regression model. Finally, considering the interrelationship between sleep, hypertension, and obstructive sleep apnea (OSA), we further adjusted for baseline OSA risk based on Berlin Questionnaire in the final model (26). All statistical analyses were performed using SAS version 9.4 (SAS Institute Inc.). A two-sided *P*-value < 0.05 was considered statistically significant.

## Results

The baseline characteristics of all participants are shown in Table 1. Among 165,493 participants, the mean age at baseline was 53.6 years (standard deviation: 8.0 years), and 37.7% were male. The proportions of participants with a healthy sleep score of 0–1, 2, 3, 4, and 5 were 3.5, 17.7, 37.7, 32.7, and 8.5%, respectively. The proportions of participants with lifestyle score of 0, 1, 2, 3, and 4 were 2.6, 21.3, 37.0, 28.8, and 10.4%, respectively. Over 1,892,157 person-years follow-up [median, 11.8 years (interquartile range: 11.0–12.5)], 10,941 incident hypertension cases were documented.

**TABLE 1:** Baseline characteristics of 165,493 participants in UK Biobank study.

Baseline characteristics	Healthy sleep score				
	0–1	2	3	4	5
Number of participants	5,861	29,280	62,321	54,043	13,988
Age, mean ± SD, y	53.7 ± 7.8	53.9 ± 7.9	54.0 ± 8.0	53.5 ± 8.1	51.9 ± 8.1
Male, *n* (%)	2,812 (48.0)	12,498 (42.7)	23,388 (37.5)	18,407 (34.1)	5,197 (37.2)
**Deprivation fifth, *n* (%)**					
First (least deprived)	946 (16.1)	5,460 (18.7)	12,436 (20.0)	11,274 (20.9)	2,925 (20.9)
Second	996 (17.0)	5,516 (18.8)	12,420 (19.9)	11,200 (20.7)	2,941 (21.0)
Third	1,118 (19.1)	5,730 (19.6)	12,438 (20.0)	10,977 (20.3)	2,796 (20.0)
Forth	1,171 (20.0)	5,893 (20.1)	12,559 (20.2)	10,671 (19.8)	2,767 (19.8)
Fifth (most deprived)	1,621 (27.7)	6,646 (22.7)	12,397 (19.9)	9,851 (18.2)	2,541 (18.2)
Missing	9 (0.2)	35 (0.1)	71 (0.1)	70 (0.1)	18 (0.1)
BMI, mean ± SD, kg/m^2^	28.0 ± 4.7	26.9 ± 4.4	26.0 ± 4.1	25.4 ± 3.8	25.1 ± 3.7
**Education, *n* (%)**					
College or university	1,809 (30.9)	10,162 (34.7)	23,821 (38.2)	22,933 (42.4)	6,453 (46.1)
Vocational	670 (11.4)	3,158 (10.8)	6,379 (10.2)	5,016 (9.3)	1,223 (8.7)
Upper secondary	674 (11.5)	3,577 (12.2)	7,900 (12.7)	6,808 (12.6)	1,811 (13.0)
Lower secondary	1,835 (31.3)	8,633 (29.5)	17,170 (27.6)	14,119 (26.1)	3,457 (24.7)
Others	840 (14.3)	3,569 (12.2)	6,711 (10.8)	4,911 (9.1)	978 (7.0)
Unknown	33 (0.6)	181 (0.6)	340 (0.6)	256 (0.5)	66 (0.5)
**Ethnicity, *n* (%)**					
White	5,411 (92.3)	27,626 (94.4)	59,236 (95.1)	51,574 (95.4)	13,295 (95.1)
Mixed	52 (0.9)	233 (0.8)	430 (0.7)	364 (0.7)	65 (0.5)
Asian	149 (2.5)	511 (1.8)	1,069 (1.7)	839 (1.6)	287 (2.1)
Black	120 (2.1)	430 (1.5)	693 (1.1)	541 (1.0)	148 (1.1)
Chinese	27 (0.5)	129 (0.4)	216 (0.4)	180 (0.3)	49 (0.4)
Others	85 (1.5)	277 (1.0)	526 (0.8)	415 (0.8)	115 (0.8)
Missing	17 (0.3)	74 (0.3)	151 (0.2)	130 (0.2)	29 (0.2)
**Household income, £, *n* (%)**					
< 18,000	1,143 (19.5)	4,758 (16.3)	8,888 (14.3)	6,795 (12.6)	1,499 (10.7)
18,000–30,999	1,219 (20.8)	6,096 (20.8)	12,558 (20.2)	10,550 (19.5)	2,438 (17.4)
31,000–51,999	1,460 (24.9)	7,231 (24.7)	15,915 (25.5)	13,789 (25.5)	3,649 (26.1)
52,000–100,000	1,096 (18.7)	6,253 (21.4)	14,100 (22.6)	13,294 (24.6)	3,775 (27.0)
> 100,000	252 (4.3)	1,646 (5.6)	4,078 (6.5)	4,037 (7.5)	1,340 (9.6)
Missing	691 (11.8)	3,296 (11.3)	6,782 (10.9)	5,578 (10.3)	1,287 (9.2)
Moderate alcohol consumption, *n* (%)	2,938 (50.1)	15,491 (52.9)	34,036 (54.6)	30,742 (56.9)	8,245 (58.9)
**Healthy lifestyle factors, *n* (%)**					
No current smoking	4,649 (79.3)	24,602 (84)	55,327 (88.8)	49,750 (92.1)	13,036 (93.2)
Regular physical activity	2,863 (48.9)	15,028 (51.3)	34,443 (55.3)	31,583 (58.4)	8,625 (61.7)
Healthy diet	1,563 (26.7)	8,616 (29.4)	20,659 (33.2)	19,574 (36.2)	5,442 (38.9)
Healthy body weight	1,607 (27.4)	10,362 (35.4)	27,131 (43.5)	26,895 (49.8)	7,458 (53.3)
**Having low-risk sleep factors, *n* (%)**					
Early chronotype	359 (6.1)	7,793 (26.6)	34,128 (54.8)	45,503 (84.2)	13,988 (100)
Sleep 7–8 h/day	267 (4.6)	10,652 (36.4)	41,681 (66.9)	50,565 (93.6)	13,988 (100)
Never/rarely insomnia	65 (1.1)	1,658 (5.7)	9,043 (14.5)	18,728 (34.7)	13,988 (100)
No self-reported snoring	318 (5.4)	10,647 (36.4)	40,711 (65.3)	47,496 (87.9)	13,988 (100)
No frequent daytime sleepiness	4,583 (78.2)	27,810 (95.0)	61,400 (98.5)	53,880 (99.7)	13,988 (100)
Systolic blood pressure, mean ± SD, mm Hg	124.8 ± 9.5	124.3 ± 9.7	123.9 ± 9.8	123.5 ± 10.0	123.2 ± 10.0
Diastolic blood pressure, mean ± SD, mm Hg	77.3 ± 6.9	76.7 ± 6.9	76.2 ± 6.9	75.8 ± 7.0	75.7 ± 7.0
Cardiovascular disease, *n* (%)	242 (4.1)	956 (3.3)	1,765 (2.8)	1,220 (2.3)	266 (1.9)
Diabetes mellitus, *n* (%)	256 (4.4)	879 (3)	1,384 (2.2)	999 (1.9)	246 (1.8)
Chronic kidney disease, *n* (%)	78 (1.3)	313 (1.1)	537 (0.9)	429 (0.8)	71 (0.5)
Cancer, *n* (%)	499 (8.5)	2,418 (8.3)	5,422 (8.7)	4,409 (8.2)	1,018 (7.3)

*SD, standard deviation; IQR, interquartile range; BMI, body mass index.*

The multivariable-adjusted HRs for hypertension by healthy sleep scores and healthy lifestyle scores are shown in Table 2. We found that a higher sleep score was associated with a lower risk of hypertension after adjusting for a wide range of covariates and lifestyle categories in Model 3. Participants with a sleep score of 5 had a fully adjusted HR of 0.58 (95% CI: 0.52, 0.65) for hypertension compared to those with a sleep score of 0–1 (*P* trend < 0.0001), and a 1 score increment in healthy sleep score was associated with a 12% lower risk of hypertension [HR, 0.88 (95% CI: 0.86, 0.90)]. Similarly, the healthy lifestyle score was inversely associated with the risk of hypertension, and the fully adjusted HR was 0.48 (95% CI: 0.43, 0.54) in participants with a lifestyle score of 4 compared with those with a score of 0 (*P* trend < 0.0001). A 1 score increment in healthy lifestyle score was associated with a 16% lower risk of hypertension [HR, 0.84 (95% CI: 0.83, 0.86)]. In terms of individual factors of sleep patterns and lifestyles, short or long sleep duration, self-reported insomnia, snoring, and excessive daytime sleepiness, as well as smoking, physical inactivity, poor-quality diet, and not optimal body weight, were all associated with the risk of hypertension in the fully adjusted model (Supplementary Tables 5, 6).

**TABLE 2:** Hazard ratios (HRs) for hypertension by healthy sleep score or healthy lifestyle score among 165,493 participants.

	Healthy sleep score					*P* for trend	Per score increment
	0–1	2	3	4	5		
Total participants	5,861	29,280	62,321	54,043	13,988		
No. of hypertension cases/person-years	647/65,239	2,416/331,474	4,318/711,482	2,954/621,914	606/162,048		
Model 1	1.00 (ref)	0.73 (0.67, 0.80)	0.61 (0.56, 0.66)	0.50 (0.45, 0.54)	0.42 (0.38, 0.47)	<0.0001	0.81 (0.80, 0.83)
Model 2	1.00 (ref)	0.79 (0.73, 0.87)	0.71 (0.65, 0.77)	0.61 (0.56, 0.66)	0.53 (0.48, 0.60)	<0.0001	0.86 (0.85, 0.88)
Model 3	1.00 (ref)	0.83 (0.76, 0.91)	0.76 (0.69, 0.82)	0.65 (0.60, 0.71)	0.58 (0.52, 0.65)	<0.0001	0.88 (0.86, 0.90)

	Healthy lifestyle score*						
	
	**0**	**1**	**2**	**3**	**4**		

Total participants	4,253	35,216	61,198	47,703	17,123		
No. of hypertension cases/person-years	480/47,198	3,009/398,324	4,250/698,536	2,479/549,542	723/198,557		
Model 1	1.00 (ref)	0.69 (0.62, 0.76)	0.55 (0.50, 0.60)	0.40 (0.36, 0.44)	0.31 (0.28, 0.35)	<0.0001	0.76 (0.74, 0.77)
Model 2	1.00 (ref)	0.76 (0.69, 0.84)	0.65 (0.59, 0.72)	0.52 (0.47, 0.57)	0.44 (0.39, 0.50)	<0.0001	0.82 (0.81, 0.84)
Model 3	1.00 (ref)	0.78 (0.70, 0.85)	0.69 (0.62, 0.75)	0.55 (0.50, 0.61)	0.48 (0.43, 0.54)	<0.0001	0.84 (0.83, 0.86)

Model 1 adjusted for age and sex; Model 2 adjusted for Model 1 + ethnicity, education, Townsend deprivation index, household income, baseline mean arterial pressure, alcohol consumption, and healthy lifestyle categories; Model 3 adjusted for Model 2 + family history of hypertension, baseline cardiovascular disease, diabetes, chronic kidney disease, and cancer. *Adjusted for healthy sleep pattern categories in Models 2 and 3. Ref, reference.

**FIGURE 1:**
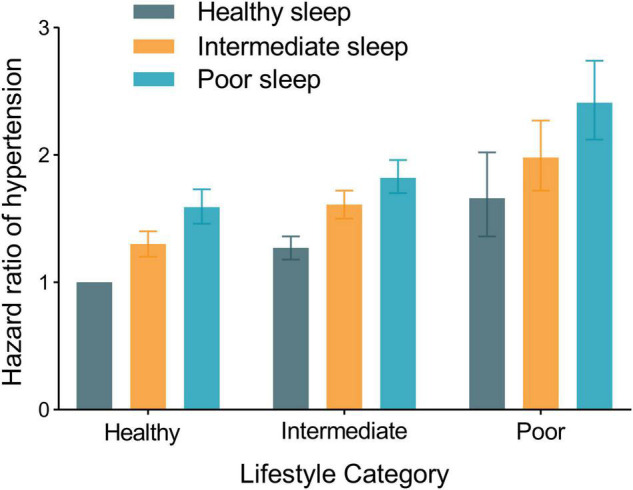
Joint association of sleep patterns and lifestyles with hypertension incidence among 165,493 participants. The models were fully adjusted for age, sex, ethnicity, education, Townsend deprivation index, household income, baseline mean arterial pressure, alcohol consumption, healthy lifestyle categories, family history of hypertension, baseline cardiovascular disease, diabetes, chronic kidney disease, and cancer.

We further examined the joint association of sleep patterns and lifestyles with the risk of hypertension (Figure 1 and Supplementary Table 7). A healthy sleep pattern within each category of lifestyle, as well as a healthy lifestyle in each sleep pattern stratum, was associated with a lower risk of hypertension (Supplementary Table 8). Compared with those who have a healthy sleep pattern and a healthy lifestyle, participants with a poor sleep pattern and a poor lifestyle had the highest risk of subsequent hypertension [adjusted HR, 2.41 (95% CI: 2.12, 2.74)], though the interaction between sleep patterns and lifestyle was not significant (*P* for interaction = 0.13).

**FIGURE 2:**
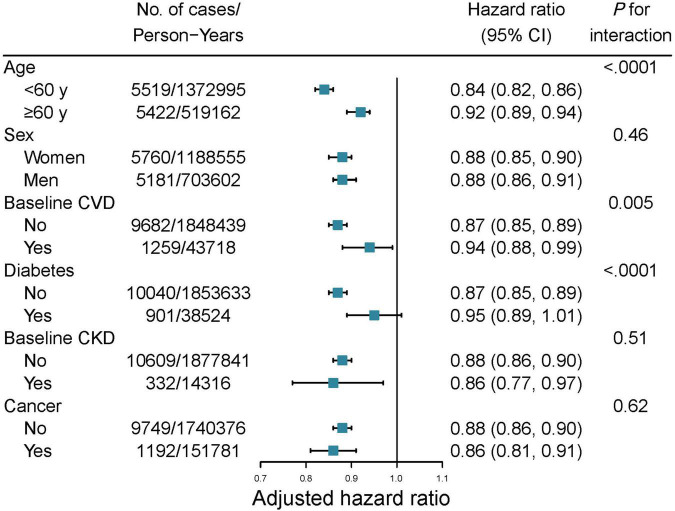
Subgroups analysis of the healthy sleep score with incident hypertension in UK Biobank. The models were fully adjusted for age, sex, ethnicity, education, Townsend deprivation index, household income, baseline mean arterial pressure, alcohol consumption, healthy lifestyle categories, family history of hypertension, baseline cardiovascular disease, diabetes, chronic kidney disease, and cancer.

We further conducted stratified analyses according to individual lifestyle factors. A higher healthy sleep score was associated with a lower risk of hypertension in all categories, whereas no significant interactions were found (all *P* for interaction > 0.05, Supplementary Figure 2). Stratified analyses were also conducted according to other potential risk factors, including age, sex, baseline diabetes, CVD, CKD, and cancer. The associations between healthy sleep score and the risk of hypertension were stronger among participants younger than 60 years (*P* for interaction < 0.0001), without CVD (*P* for interaction = 0.005), and without diabetes at baseline (*P* for interaction < 0.0001; Figure 2).

**FIGURE 3:**
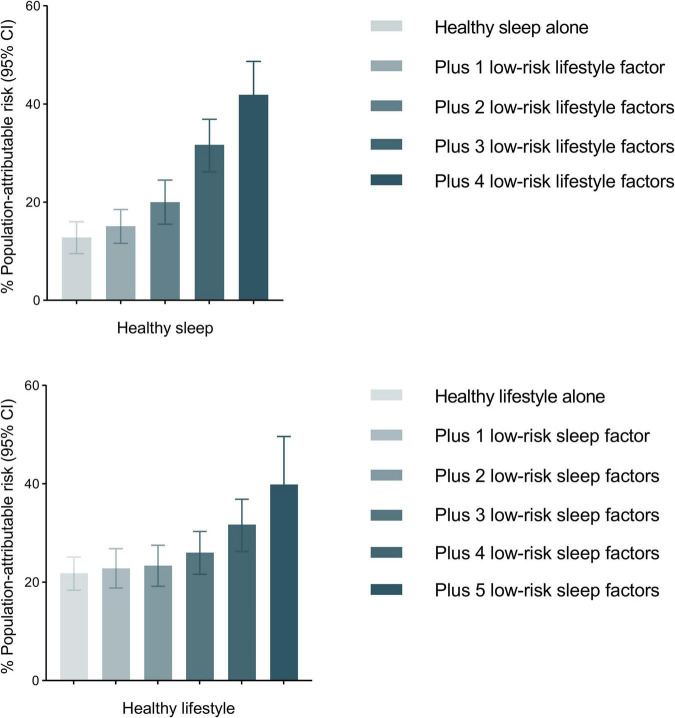
PAR estimates for hypertension incidence associated with sleep scores and lifestyle scores. PARs and 95% CIs were calculated adjusting for age, sex, ethnicity, education, Townsend deprivation index, household income, baseline mean arterial pressure, alcohol consumption status, family history of hypertension, baseline cardiovascular disease, diabetes, chronic kidney disease, and cancer; Healthy sleep is defined as healthy sleep score ≥ 4; Healthy lifestyle is defined as healthy lifestyle score ≥ 3.

We also calculated the PAR% for healthy sleep patterns and healthy lifestyles (Supplementary Table 9). The PAR% for poor adherence to a healthy lifestyle (defined as lifestyle score ≥ 3) was 20.1% (95% CI: 17.6%, 22.6%), which increased to 31.7% (95% CI: 27.6%, 35.6%) when combined with a healthy sleep pattern. For individual factors, self-reported insomnia and overweight were top contributors to hypertension risk in sleep patterns and lifestyles, respectively (Supplementary Table 10). We further observed that PAR% increased with the increment in sleep or lifestyle score on the basis of a healthy lifestyle or a healthy sleep pattern (Figure 3 and Supplementary Table 9).

In terms of weighted sleep score instead of primary score, participants with a higher weighted score had a lower risk of hypertension, and the same results pattern was observed when the weighted score was categorized into quintiles (Supplementary Table 11). Additionally, when combined with lifestyle categories, the joint association of the weighted sleep pattern and lifestyle with the hypertension risk did not change materially (Supplementary Table 12).

In the sensitivity analyses, the results remained largely unchanged when additionally adjusted for lipid-lowering medication use and diabetes medication use (Supplementary Table 13, Model 2). The association of healthy sleep score and lifestyle score with hypertension risk was lightly attenuated but still significant after further adjusting for baseline OSA risk (Supplementary Table 13, Model 3). We repeated our analyses after excluding participants with a hypertension event within the first 2 years of follow-up or those with self-reported CVD at recruitment, and the associations of sleep patterns and lifestyles with the hypertension risk were still robust (Supplementary Tables 14, 15). In addition, similar results were observed regarding the association of the joint effects of sleep patterns and lifestyles with the risk of hypertension (Supplementary Tables 16, 17).

## Discussion

In this study of 165,493 participants from the UK Biobank, we observed that adherence to a healthy sleep pattern and a healthy lifestyle was independently associated with a decreased risk of subsequent hypertension. Participants with both a poor sleep pattern and a poor lifestyle had a 2.41-fold risk of hypertension compared with those who kept a healthy sleep pattern and lifestyle. Though speculative, if these associations were causal, approximately 14.7, 20.1, and 31.7% of hypertension cases would be avoided when all participants had a healthy sleep pattern, a healthy lifestyle, or both, respectively.

Previous studies have discussed the role of sleep behaviors in the development of hypertension. Although the majority of prior evidence was consistent with our findings, several results were conflicting. Meta-analyses found that short sleep duration but not long sleep was associated with the risk of hypertension (13, 27, 28). Nonetheless, we observed that not only short sleep but somewhat long sleep duration was significantly associated with subsequent hypertension, which might need further evidence to clarify the relationship between long sleep duration and hypertension incidence. Insomnia was another widely investigated sleep factor for hypertension. Consistent with previous studies, we observed a significant association between self-reported insomnia and hypertension incidence in this middle-aged population (16, 29, 30). Previous studies have also shown the associations of snoring (15), chronotype (31), and excessive daytime sleepiness (32) with hypertension. However, few prospective studies have investigated the association between the combination of these sleep parameters and the risk of hypertension or focused on insomnia and sleep duration only (16, 29, 33). The Penn State study on 786 individuals showed that chronic insomniacs with short sleep duration (< 6 h) had the highest risk of hypertension compared with normal sleepers who slept ≥ 6 h [adjusted odds ratio, 3.8 (95% CI: 1.6, 9.0)] after 7.5 years of follow-up (16). A recent study on 2,148 US Latinos observed the association of actigraphy-based sleep duration, sleep fragmentation index, sleep efficiency, insomnia, and frequent napping with the prevalence of hypertension (34). This study gave a hint that a combination of different sleep domains might be associated with hypertension, however, the cross-sectional study design and the limited sample size hampered the interpretation and generalization of the study. Moreover, the pathophysiological pathways involved in the impacts of sleep behaviors on hypertension and other cardiometabolic dysfunctions might be overlapped (35), that mainly included systemic inflammation (36), vascular endothelial dysfunction and oxidative stress (37), stimulation of the renin-angiotensin-aldosterone system (38), and altered tone of the sympathetic nervous system (39), the latter of which might also impact the nighttime blood pressure and cause the non-dipping pattern (40). This mechanistic evidence underscored the possible synergistic effects and the multi-dimensional influence of sleep disorders on the occurrence and development of CVDs including hypertension. These findings indicated that considering sleep parameters in combination is more practical in the related studies. Meanwhile, the large sample size and the prospective study design further strengthened our results.

Healthy lifestyle factors, including not current smoking, regular physical exercise, maintaining healthy body weight, and having a healthy diet are highly recommended in the ESC/ESH and American Heart Association guidelines for hypertension prevention and management (7, 8). However, evidence on the temporal association between healthy lifestyle defined by these four factors and the risk of hypertension was still limited. In the Nurses’ Health Study II, adherence to a healthy lifestyle with three low-risk factors [BMI < 25, daily vigorous exercise > 30 min, and Dietary Approaches to Stop Hypertension (DASH) scores in the top quintile] was associated with a low risk of self-reported hypertension, and the PAR% was 53% (95% CI: 45%, 60%) (9), which is higher than the PAR% for adherence to more than three healthy lifestyle factors in our study [20.1% (95% CI:17.6%, 22.6%)]. The difference might be explained by the younger (mean age, 36 years) female population, longer follow-up (14 years), and the specific definition of low-risk lifestyle factors in the Nurses’ Health Study II, because hypertension is progressively prevalent with increased age and more common in males (41). The Jackson Heart study in 1,878 blacks found that adherence to three of four health behaviors in Life’s simple 7 (BMI, physical activity, diet, and smoking status) was associated with a 60% reduction in hypertension risk (HR: 0.40, 95% CI: 0.25, 0.64) after a median of 8.0 years of follow-up (42), which is close to the 52% reduction of hypertension risk (HR: 0.48, 95% CI: 0.43, 0.54) observed in our analysis. Our findings added strong evidence to the association of lifestyle with the risk of hypertension and emphasized the importance of adherence to an overall healthy lifestyle for hypertension prevention.

Sleep is increasingly recognized as a crucial lifestyle contributor to hypertension and other cardiometabolic health (43). However, evidence on the effect of sleep in conjunction with lifestyle on hypertension incidence is sparse. A prospective study conducted among adults with hypertension in the Tongji-Dongfeng cohort found that adopting a healthy lifestyle (constructed by BMI, diet, physical activity, smoking, and sleep duration) is associated with benefits in the prevention of premature death among hypertensives using or not using antihypertensive medication (44). This study showed that a healthy lifestyle including a sleep parameter plays an important role in the management of hypertension, which in some way supported our findings. In our study, we observed that a healthy sleep pattern was consistently associated with a lower risk of hypertension independent of lifestyle strata, and a healthy lifestyle was similarly related to a lower risk of hypertension in each sleep stratum. Furthermore, the great benefit of adherence to a healthy lifestyle and the incremental benefit after combining a healthy sleep pattern with a healthy lifestyle indicates that a healthy lifestyle is the cornerstone of hypertension prevention, whereas adherence to a healthy sleep pattern might complement the well-established lifestyle for the primary prevention of hypertension. Although sleep duration and some sleep disorders have been reported associated with hypertension risk in scientific statements (43), the present guidelines only recommend the following lifestyle factors for the primary prevention of hypertension: weight control, increased physical activity, smoking cessation, alcohol moderation, and healthy diet (7). Though more high-quality studies are warranted to incorporate these modifiable sleep factors in the recommendation of hypertension prevention, our findings provided robust evidence that adherence to a healthy sleep pattern exerts protection against hypertension regardless of previously recommended lifestyles.

The prospective study design and the large sample size are the two main strengths of this study. Another strength is that we constituted an overall sleep pattern using five sleep behaviors, which describes sleep conditions more comprehensively. Moreover, to our knowledge, this is the first longitudinal study to estimate the joint association of sleep pattern and lifestyle with incident hypertension. Our study has several limitations. First, sleep parameters and part of lifestyles were self-reported through simplified questionnaires in this large-population-based study rather than objectively measured or physicians diagnosed (for insomnia), therefore, recall bias and misclassification of exposures were possible. However, misclassifications would likely bias our estimates toward the null. Second, the healthy sleep score did not include other sleep factors, such as periodic limb movement disorder and restless leg syndrome, which might be involved in the occurrence and development of hypertension (43). Third, approximately 19% of participants missed data on any sleep parameters. However, those excluded from our analyses for missing data on sleep broadly had similar baseline characteristics to those included and had a higher incidence of hypertension (Supplementary Table 18). Therefore, the association of the sleep pattern and hypertension risk in our study might be underestimated. Fourth, although individual sleep and lifestyle factors were differentially associated with the risk of hypertension, we assigned equal weight to each sleep and lifestyle factor in our healthy sleep pattern score and healthy lifestyle score. However, our analyses based on weighted scores yielded similar results. Fifth, though moderate alcohol consumption was recommended in hypertension prevention guidelines, we did not include alcohol consumption in the overall lifestyle because of the increasing controversies over the health effects of alcohol consumption, as well as the possible inaccurate estimate of alcohol intake in the UK Biobank (45). Therefore, we treated alcohol drinking status as a confounder in this analysis. Sixth, the interpretation of PAR% assumes a causal relationship, whereas inferences on causality should be made with caution due to the observational nature of this study. Seventh, we considered a wide range of confounders, but the residual confounding from unmeasured or unknown factors might remain. Additionally, the participants were predominantly White, which limits the generalizability of our findings to other racial or ethnic groups. However, the relative homogeneity of this study population reduces confounding and potential bias. Finally, reverse causality might exist in our study, although we strictly excluded participants at baseline, and the results remained unchanged after excluding participants with a hypertension event during the first 2 years of follow-up.

## Conclusion

Our findings indicated that adherence to a healthy sleep pattern and a healthy lifestyle is associated with a low risk of hypertension, and the benefits of adhering to a healthy sleep pattern complement the well-established lifestyle for the optimal primary prevention of hypertension. These findings suggest that a healthy sleep pattern is an important part of a healthful and productive lifestyle for hypertension prevention.

## Data Availability Statement

Publicly available datasets were analyzed in this study. This data can be found here: www.ukbiobank.ac.uk/register-apply.

## Ethics Statement

The studies involving human participants were reviewed and approved by the North West Multi-center Research Ethics Committee (REC reference for UK Biobank 11/NW/0382). The patients/participants provided their written informed consent to participate in this study.

## Author Contributions

LC had full assess to all of the data in the study and took responsibility for the integrity of the data and the accuracy of the data analysis. LL was the supervisor of this study. LC and LL conceived and designed the study. YL and LC were in charge of the statistical analyses and had primary responsibility for writing the manuscript. LC, GJ, XT, and WB critically reviewed the manuscript for important intellectual content. All authors approved the final version.

## Conflict of Interest

The authors declare that the research was conducted in the absence of any commercial or financial relationships that could be construed as a potential conflict of interest.

## Publisher’s Note

All claims expressed in this article are solely those of the authors and do not necessarily represent those of their affiliated organizations, or those of the publisher, the editors and the reviewers. Any product that may be evaluated in this article, or claim that may be made by its manufacturer, is not guaranteed or endorsed by the publisher.

## Footnotes

1
www.ukbiobank.ac.uk

## Abbreviations

BMI: Body mass index
CKD: Chronic kidney disease
CVD: Cardiovascular disease
DASH: Dietary Approaches to Stop Hypertension
DBP: Diastolic blood pressure
ICD: International Classification of Diseases
MAP: Mean arterial pressure
PAR: Population attributable risk
SBP: Systolic blood pressure
SSB: Sugar-sweetened beverage.

## References

[1] Virani SS Alonso A Benjamin EJ Bittencourt MS Callaway CW Carson AP Heart disease and stroke statistics-2020 update: a report from the american heart association. *Circulation.* (2020) 141:139e–596e. 10.1161/CIR.0000000000000757 31992061

[2] Leigh J Rafiee A Oancea B Tantawi ME. Global burden of 87 risk factors in 204 countries and territories, 1990-2019: a systematic analysis for the Global Burden of Disease Study 2019. *Lancet.* (2020) 396:1223–49. 33069327 10.1016/S0140-6736(20)30752-2 PMC7566194

[3] Bowman TS Gaziano JM Buring JE Sesso HD. A prospective study of cigarette smoking and risk of incident hypertension in women. *J Am Coll Cardiol.* (2007) 50:2085–92. 10.1016/j.jacc.2007.08.017 18021879

[4] Lee JY Ryu S Sung KC. Association of baseline level of physical activity and its temporal changes with incident hypertension and diabetes mellitus. *Eur J Prevent Cardiol.* (2018) 25:1065–73. 10.1177/2047487318774419 29719968

[5] Shihab HM Meoni LA Chu AY Wang NY Ford DE Liang KY Body mass index and risk of incident hypertension over the life course: the johns hopkins precursors study. *Circulation.* (2012) 126:2983–9. 10.1161/CIRCULATIONAHA.112.117333 23151344 PMC3743236

[6] Lelong H Blacher J Baudry J Adriouch S Galan P Fezeu L Individual and combined effects of dietary factors on risk of incident hypertension: prospective analysis from the nutrinet-santé cohort. *Hypertension.* (2017) 70:712–20. 10.1161/HYPERTENSIONAHA.117.09622 28760943

[7] Williams B Mancia G Spiering W Agabiti Rosei E Azizi M Burnier M 2018 ESC/ESH Guidelines for the management of arterial hypertension. *Eur Heart J.* (2018) 39:3021–104. 30165516 10.1093/eurheartj/ehy339

[8] Whelton PK Carey RM Aronow WS Casey DE Jr. Collins KJ Dennison Himmelfarb C 2017 ACC/AHA/AAPA/ABC/ACPM/AGS/APhA/ASH/ASPC/NMA/PCNA guideline for the prevention, detection, evaluation, and management of high blood pressure in adults: a report of the american college of cardiology/american heart association task force on clinical practice guidelines. *J Am Coll Cardiol.* (2018) 71:e127–248. 29146535 10.1016/j.jacc.2017.11.006

[9] Forman JP Stampfer MJ Curhan GC. Diet and lifestyle risk factors associated with incident hypertension in women. *JAMA.* (2009) 302:401–11. 10.1001/jama.2009.1060 19622819 PMC2803081

[10] Plante TB Koh I Judd SE Howard G Howard VJ Zakai NA Life’s simple 7 and incident hypertension: the regards study. *J Am Heart Assoc.* (2020) 9:e016482. 10.1161/JAHA.120.016482 32928039 PMC7792383

[11] Lelong H Blacher J Baudry J Adriouch S Galan P Fezeu L Combination of healthy lifestyle factors on the risk of hypertension in a large cohort of French adults. *Nutrients.* (2019) 11:1687. 10.3390/nu11071687 31340445 PMC6683281

[12] Li X Sotres-Alvarez D Gallo LC Ramos AR Aviles-Santa L Perreira KM Associations of sleep disordered breathing and insomnia with incident hypertension and diabetes: the hispanic community health study/study of latinos. *Am J Respir Crit Care Med.* (2020) 203:356–65. 10.1164/rccm.201912-2330oc 32758008 PMC7874314

[13] Jike M Itani O Watanabe N Buysse DJ Kaneita Y. Long sleep duration and health outcomes: a systematic review, meta-analysis and meta-regression. *Sleep Med Rev.* (2018) 39:25–36. 10.1016/j.smrv.2017.06.011 28890167

[14] Lieu SJ Curhan GC Schernhammer ES Forman JP. Rotating night shift work and disparate hypertension risk in African-Americans. *J Hypertens.* (2012) 30:61–6. 10.1097/hjh.0b013e32834e1ea3 22134389

[15] Lee SK Choi K Chang YH Kim J Shin C. Increased risk for new-onset hypertension in midlife male snorers: the 14-year follow-up study. *J Sleep Res.* (2019) 28:e12757. 10.1111/jsr.12757 30252172

[16] Fernandez-Mendoza J Vgontzas AN Liao D Shaffer ML Vela-Bueno A Basta M Insomnia with objective short sleep duration and incident hypertension: the Penn State Cohort. *Hypertension.* (2012) 60:929–35. 10.1161/hypertensionaha.112.193268 22892811 PMC3679545

[17] Sudlow C Gallacher J Allen N Beral V Burton P Danesh J UK Biobank: an open access resource for identifying the causes of a wide range of complex diseases of middle and old age. *PLoS Med.* (2015) 12:e1001779. 10.1371/journal.pmed.1001779 25826379 PMC4380465

[18] Fan M Sun D Zhou T Heianza Y Lv J Li L Sleep patterns, genetic susceptibility, and incident cardiovascular disease: a prospective study of 385 292 UK biobank participants. *Eur Heart J.* (2019) 41:1182–9. 10.1093/eurheartj/ehz849 31848595 PMC7071844

[19] Jiao L Mitrou PN Reedy J Graubard BI Hollenbeck AR Schatzkin A A combined healthy lifestyle score and risk of pancreatic cancer in a large cohort study. *Arch Intern Med.* (2009) 169:764–70. 10.1001/archinternmed.2009.46 19398688 PMC3498842

[20] Piercy KL Troiano RP Ballard RM Carlson SA Fulton JE Galuska DA The physical activity guidelines for americans. *JAMA.* (2018) 320:2020–8. 30418471 10.1001/jama.2018.14854 PMC9582631

[21] Said MA Verweij N van der Harst P. Associations of combined genetic and lifestyle risks with incident cardiovascular disease and diabetes in the UK biobank study. *JAMA Cardiol.* (2018) 3:693–702. 10.1001/jamacardio.2018.1717 29955826 PMC6143077

[22] Mozaffarian D. Dietary and policy priorities for cardiovascular disease, diabetes, and obesity: a comprehensive review. *Circulation.* (2016) 133:187–225. 10.1161/CIRCULATIONAHA.115.018585 26746178 PMC4814348

[23] Wuopio J Orho-Melander M Ärnlöv J Nowak C. Estimated salt intake and risk of atrial fibrillation in a prospective community-based cohort. *J Intern Med.* (2021) 289:700–8. 10.1111/joim.13194 33210391 PMC8246952

[24] Zhong VW Van Horn L Cornelis MC Wilkins JT Ning H Carnethon MR Associations of dietary cholesterol or egg consumption with incident cardiovascular disease and mortality. *JAMA.* (2019) 321:1081–95. 10.1001/jama.2019.1572 30874756 PMC6439941

[25] Spiegelman D Hertzmark E Wand HC. Point and interval estimates of partial population attributable risks in cohort studies: examples and software. *Cancer Causes Control.* (2007) 18:571–9. 10.1007/s10552-006-0090-y 17387622

[26] Tan X Benedict C. Sleep characteristics and HbA1c in patients with type 2 diabetes on glucose-lowering medication. *BMJ Open Diab Res Care.* (2020) 8:e001702. 10.1136/bmjdrc-2020-001702 32868313 PMC7462247

[27] Wang Q Xi B Liu M Zhang Y Fu M. Short sleep duration is associated with hypertension risk among adults: a systematic review and meta-analysis. *Hypertens Res.* (2012) 35:1012–8. 10.1038/hr.2012.91 22763475

[28] Knutson KL Van Cauter E Rathouz PJ Yan LL Hulley SB Liu K Association between sleep and blood pressure in midlife: the CARDIA sleep study. *Arch Intern Med.* (2009) 169:1055–61. 10.1001/archinternmed.2009.119 19506175 PMC2944774

[29] Vgontzas AN Liao D Bixler EO Chrousos GP Vela-Bueno A. Insomnia with objective short sleep duration is associated with a high risk for hypertension. *Sleep.* (2009) 32:491–7. 10.1093/sleep/32.4.491 19413143 PMC2663863

[30] Dong Y Yang FM. Insomnia symptoms predict both future hypertension and depression. *Prev Med.* (2019) 123:41–7. 10.1016/j.ypmed.2019.02.001 30742871

[31] Merikanto I Lahti T Puolijoki H Vanhala M Peltonen M Laatikainen T Associations of chronotype and sleep with cardiovascular diseases and type 2 diabetes. *Chronobiol Int.* (2013) 30:470–7. 10.3109/07420528.2012.741171 23281716

[32] Endeshaw Y Rice TB Schwartz AV Stone KL Manini TM Satterfield S Snoring, daytime sleepiness, and incident cardiovascular disease in the health, aging, and body composition study. *Sleep.* (2013) 36:1737–45. 10.5665/sleep.3140 24179308 PMC3792392

[33] Kalmbach DA Pillai V Arnedt JT Drake CL. DSM-5 insomnia and short sleep: comorbidity landscape and racial disparities. *Sleep.* (2016) 39:2101–11. 10.5665/sleep.6306 27634805 PMC5103798

[34] Ramos AR Weng J Wallace DM Petrov MR Wohlgemuth WK Sotres-Alvarez D Sleep patterns and hypertension using actigraphy in the hispanic community health study/study of latinos. *Chest.* (2018) 153:87–93. 10.1016/j.chest.2017.09.028 28970105 PMC5812757

[35] Valenzuela PL Carrera-Bastos P Gálvez BG Ruiz-Hurtado G Ordovas JM Ruilope LM Lifestyle interventions for the prevention and treatment of hypertension. *Nat Rev Cardiol.* (2020) 18:251–75. 33037326 10.1038/s41569-020-00437-9

[36] Irwin MR Olmstead R Carroll JE. Sleep disturbance, sleep duration, and inflammation: a systematic review and meta-analysis of cohort studies and experimental sleep deprivation. *Biol Psychiat.* (2016) 80:40–52. 10.1016/j.biopsych.2015.05.014 26140821 PMC4666828

[37] Faraut B Boudjeltia KZ Vanhamme L Kerkhofs M. Immune, inflammatory and cardiovascular consequences of sleep restriction and recovery. *Sleep Med Rev.* (2012) 16:137–49. 10.1016/j.smrv.2011.05.001 21835655

[38] Thiel S Haile SR Peitzsch M Schwarz EI Sievi NA Kurth S Endocrine responses during CPAP withdrawal in obstructive sleep apnoea: data from two randomised controlled trials. *Thorax.* (2019) 74:1102–5. 10.1136/thoraxjnl-2019-213522 31467191

[39] Tobaldini E Cogliati C Fiorelli EM Nunziata V Wu MA Prado M One night on-call: sleep deprivation affects cardiac autonomic control and inflammation in physicians. *Eur J Intern Med.* (2013) 24:664–70. 10.1016/j.ejim.2013.03.011 23601527

[40] Sherwood A Steffen PR Blumenthal JA Kuhn C Hinderliter AL. Nighttime blood pressure dipping: the role of the sympathetic nervous system. *Am J Hypertens.* (2002) 15:111–8. 10.1016/s0895-7061(01)02251-8 11863245

[41] Chow CK Teo KK Rangarajan S Islam S Gupta R Avezum A Prevalence, awareness, treatment, and control of hypertension in rural and urban communities in high-, middle-, and low-income countries. *JAMA.* (2013) 310:959–68. 10.1001/jama.2013.184182 24002282

[42] Booth JN III Abdalla M Tanner RM Diaz KM Bromfield SG Tajeu GS Cardiovascular Health and Incident Hypertension in Blacks: JHS (The Jackson Heart Study). *Hypertension.* (2017) 70:285–92. 10.1161/hypertensionaha.117.09278 28652461 PMC5823255

[43] St-Onge MP Grandner MA Brown D Conroy MB Jean-Louis G Coons M Sleep duration and quality: impact on lifestyle behaviors and cardiometabolic health: a scientific statement from the american heart association. *Circulation.* (2016) 134:e367–86. 10.1161/CIR.0000000000000444 27647451 PMC5567876

[44] Lu Q Zhang Y Geng T Yang K Guo K Min X Association of lifestyle factors and antihypertensive medication use with risk of all-cause and cause-specific mortality among adults with hypertension in China. *JAMA Netw Open.* (2022) 5:e2146118. 10.1001/jamanetworkopen.2021.46118 35103793 PMC8808332

[45] Xue A Jiang L Zhu Z Wray NR Visscher PM Zeng J Genome-wide analyses of behavioural traits are subject to bias by misreports and longitudinal changes. *Nat Commun.* (2021) 12:20211. 10.1038/s41467-020-20237-6 PMC7804181 33436567

