# Supplementary material for: Association of Sleep Patterns and Lifestyles With Incident Hypertension: Evidence From a Large Population-Based Cohort Study
Source: Front Cardiovasc Med. 2022 Apr 1;9:847452. doi: 10.3389/fcvm.2022.847452 (PMC9010545; doi:10.3389/fcvm.2022.847452)
Supplement: Supplementary file 1 [file Data_Sheet_1.pdf]

Supplemental Online Content

Table S1. ICD code and Field ID used in disease definition in UK Biobank

Table S2. Definitions and Field ID used for each component of a healthy sleep pattern

Table S3. Definitions and Field ID used for each component of a healthy lifestyle

Table S4. Definitions and Field ID used for each item of a healthy diet score

Table S5. Hazard ratios (HRs) for hypertension by sleep pattern components among 165,493 participants in UK Biobank

Table S6. Hazard ratios (HRs) for hypertension by lifestyle components among 165,493 participants in the UK Biobank study

Table S7. Joint association of sleep pattern and lifestyle with hypertension incidence among 165,493 participants

Table S8. Stratified analyses by categories of sleep or lifestyle with incident hypertension among 165,493 participants

Table S9. PAR estimates for hypertension incidence associated with sleep scores and lifestyle scores separately and in combination

Table S10. PAR estimates for hypertension incidence associated with sleep scores and lifestyle scores components

Table S11. Association of weighted healthy sleep score with incident hypertension among 165,493 participants

Table S12. Joint association of weighted sleep pattern and lifestyle with incident hypertension among 165,493 participants

Table S13. Sensitive analysis (N=165,493) for the association of healthy sleep score and healthy lifestyle score with incident hypertension

Table S14. Sensitive analysis (N=164,192) for the association of healthy sleep score and healthy lifestyle score with incident hypertension after excluding individuals with hypertension events in the first 2 years of follow-up

Table S15. Sensitive analysis (N=161,044) for the association of healthy sleep score and healthy lifestyle score with incident hypertension after excluding individuals with cardiovascular diseases at baseline

Table S16. Joint association of sleep pattern and lifestyle with incident hypertension after excluding individuals with cardiovascular diseases at baseline (N=161,044)

Table S17. Joint association of sleep pattern and lifestyle with incident hypertension after excluding individuals with hypertension events in the first 2 years of follow-up (N=164,192)

Table S18. Cohort characteristics for participants with full or missing data on sleep pattern

Figure S1. Flowchart of participants enrolment.

Figure S2. Associations of per healthy sleep score increment with incident hypertension stratified by lifestyle components among 165,493 participants.

Table S1. ICD code and Field ID used in disease definition

	ICD-9	ICD-10	OPCS-4	Self-reported fields	Examination
**Hypertension**	401, 402, 403, 404, 405	I10, I11, I12, I13, I15, O10, O11		6150, 2966, 6153, 6177, 20002	4079, 4080, 93, 94
**Cardiovascular Disease**	410, 411, 412, 413, 414, 428, 429, 430, 431, 434, 436	I20-I25, I48, I50, I60, I61, I63, I64	K40-K46, K49, K50, K75, K621, K622, K623, A052-A054, L351, L353, L343	6150, 3894, 4056, 20004, 20002	
**Diabetes Mellitus**	250	E10-E14		2443, 2976, 6153, 6177, 20002	30740, 30750
**Chronic Kidney Disease**	582, 583, 585, 586, 588, V420, V56	I120, I131, N03, N05, N18, N19, N25, Z940, Z992			30700, 30720*
**Cancer at baseline**				20001, 40005	

*Baseline serum level of creatinine and cystatin C, which were used to calculate eGFR based on CKD-EPI 2012 creatinine-cystatin C equation. Participants with an eGFR value less than 60 mL/min/1.73m^2 were also treated as having chronic kidney disease at baseline.

Abbreviations: ICD, International Classification of Diseases; OPCS, Office of Population Censuses and Surveys Classification of Interventions and Procedures; eGFR, estimates of glomerular filtration rate; CKD-EPI, the Chronic Kidney Disease Epidemiology Collaboration.

Table S2. Definitions and Field ID used for each component of a healthy sleep pattern

Item	Description	Definitions of low-risk factors	Field ID
Morning/evening person (chronotype)	Chronotype was defined as four categories for participants to choose: “Definitely a ‘morning’ person”, “More a ‘morning’ than ‘evening’ person”, “More an ‘evening’ than a ‘morning’ person” and “Definitely an ‘evening’ person” in UK biobank.	“Definitely a ‘morning’ person” and “More a ‘morning’ than ‘evening’ person” were classified as an ideal chronotype in this analysis.	1180
Sleeplessness / insomnia	Touchscreen question “Do you have trouble falling asleep at night or do you wake up in the middle of the night?” “Never/rarely” was defined as not insomnia while “Sometimes” and “Usually” were insomnia.	Answer with “Never/rarely” was defined as not insomnia (ideal), “Sometimes” and “Usually” were insomnia.	1200
Snoring	Touchscreen question “Does your partner or a close relative or friend complain about your snoring?”	Answer with “No” was defined as not snoring (ideal), “Yes” was as snoring.	1210
Daytime dozing /sleeping (narcolepsy)	Touchscreen question “How likely are you to doze off or fall asleep during the daytime when you don’t mean to? (e.g. when working, reading, or driving)”	Answers with “Never/rarely” or “Sometimes” were defined as no daytime sleeping (ideal), “Often” was as daytime sleeping.	1220
Sleep duration	Touchscreen question “About how many hours sleep do you get in every 24 hours? (please include naps)”	7-8 hours were defined as the ideal length of sleep duration, less than 7 or more than 8 hours were all classified as not ideal sleep duration in this analysis.	1160

Table S3. Definitions and Field ID used for each component of a healthy lifestyle

**Low-risk lifestyle factor**	**Definition**	**Field**
No current smoking	UK Biobank Touchscreen questionnaire at baseline; Smoking status is defined as current, previous, and never smoker	20116
Regular physical activity	≥150 min/week of moderate activity OR<br>≥75 min/week of vigorous activity OR<br>≥150 min/week of moderate and vigorous activity	884, 894, 904, 914
Healthy diet	At least 4 of the following 10 food groups:<br>1. Fruits: ≥3 servings/day<br>2. Vegetables: ≥3 servings/day<br>3. Whole grains: ≥3 servings/day<br>4. Vegetable oil: ≥2 servings/day<br>5. Fish: ≥2 servings/week<br>6. Dairy: ≥2 servings/day<br>7. Refined grain: ≤2 servings/day<br>8. Unprocessed meats: ≤2 servings/week<br>9. Processed meats: ≤1 servings/week<br>10. Daily sodium intake: <2300 mg/day	1309, 1319, 1289, 1299, 1438, 1448, 1458, 1468, 1428, 1438, 2654, 1329, 1339, 1408, 1418, 1359, 1369, 1379, 1389, 1349, 30530
Healthy body weight	18.5 ≤BMI <25 kg/m^2	21001

Table S4. Definitions and Field ID used for each item of a healthy diet score

	**Goal (1 point)**	**One serving equals to**	**Field**
Fruits	≥3 servings/day	1 piece of fresh fruit<br>5 pieces of dried fruit	1309, 1319
Vegetables<br>(excluding potatoes)	≥3 servings/day	3 heaped tablespoons	1289, 1299
Whole grains	≥3 servings/day	1 slice of whole-grain bread<br>1 cup of whole-grain cereal	1438, 1448, 1458, 1468
Vegetable oil	≥2 servings/day	Vegetable oil-based spread (Flora Pro-Active/Benecol, Soft (tub) margarine, Olive oil-based spread, or Polyunsaturated/sunflower oil-based spread) in combination with eating at least 2 slices of bread	1428, 2654, 1438
Fish	≥2 servings/week	Once/week	1329, 1339
Dairy	≥2 servings/day	1 cup/day if consumption any type of milk<br>1 piece of cheese	1408, 1418
Refined grains	≤2 servings/day	1 slice of bread<br>1 bowl of cereal	1438, 1448, 1458, 1468
Unprocessed meats	≤2 servings/week	Once/week (including poultry, beef, lamb, and pork)	1359, 1369, 1379, 1389
Processed meats	≤1 servings/week	Once/week	1349
Daily sodium intake	<2300 mg/day		30530

Table S5. Hazard ratios (HRs) for hypertension by sleep pattern components among 165,493 participants in UK Biobank

	Total No. of participants	No. of cases/ Person-Years	Model 1	Model 2	Model 3	Model 4
**Chronotype**						
Late	63,722	4,165/728,645	1.00 (ref)	1.00 (ref)	1.00 (ref)	1.00 (ref)
Early	101,771	6,776/1,163,512	**0.93 (0.89, 0.96)**	**0.95 (0.92, 0.99)**	**0.96 (0.92, 0.999)**	0.96 (0.93, 1.002)
**Sleep duration**						
Not ideal (≤6 or ≥9 hours)	48,340	3,943/546,216	1.00 (ref)	1.00 (ref)	1.00 (ref)	1.00 (ref)
Ideal (7-8 hours)	117,153	6,998/1,345,942	**0.75 (0.72, 0.78)**	**0.82 (0.79, 0.86)**	**0.84 (0.81, 0.88)**	**0.86 (0.82, 0.89)**
**Insomnia**						
Sometimes/Usually	122,011	8,667/1,391,193	1.00 (ref)	1.00 (ref)	1.00 (ref)	1.00 (ref)
Never/rarely	43,482	2,274/500,964	**0.77 (0.74, 0.81)**	**0.80 (0.76, 0.84)**	**0.81 (0.78, 0.85)**	**0.83 (0.79, 0.87)**
**Snoring**						
Yes	52,333	4,273/594,606	1.00 (ref)	1.00 (ref)	1.00 (ref)	1.00 (ref)
No	113,160	6,668/1,297,551	**0.80 (0.77, 0.83)**	**0.87 (0.83, 0.90)**	**0.88 (0.85, 0.92)**	**0.88 (0.85, 0.92)**
**Excessive daytime sleepiness**						
Often/Always	3,832	428/42,441	1.00 (ref)	1.00 (ref)	1.00 (ref)	1.00 (ref)
Never/rarely/sometimes	161,661	10,513/1,849,716	**0.63 (0.58, 0.70)**	**0.69 (0.63, 0.76)**	**0.73 (0.66, 0.80)**	**0.76 (0.69, 0.84)**

Model 1 adjusted for age and sex;

Model 2 adjusted for Model 1 +ethnicity, education, Townsend deprivation index, household income, baseline mean arterial pressure, alcohol consumption status, and healthy lifestyle categories;

Model 3 adjusted for Model 2 +family history of hypertension, lipid-lowering medication use, diabetes medication use, baseline cardiovascular disease, diabetes, chronic kidney disease, and cancer;

Model 4 adjusted for confounders in Model 3 and included all low-risk sleep factors simultaneously and mutually adjusted.
Abbreviations: ref, reference.

Table S6. Hazard ratios (HRs) for hypertension by lifestyle components among 165,493 participants in the UK Biobank study

	Total No. of participants	No. of cases/ Person-Years	Model 1	Model 2	Model 3	Model 4
**Smoking status**						
Not ideal (Current)	18,129	1,630/203,228	1.00 (ref)	1.00 (ref)	1.00 (ref)	1.00 (ref)
Ideal (Never/Previous)	147,364	9,311/1,688,929	**0.62 (0.59, 0.66)**	**0.72 (0.68, 0.76)**	**0.73 (0.69, 0.77)**	**0.72 (0.68, 0.76)**
**Regular physical activity***						
Not ideal	72,951	5,257/831,965	1.00 (ref)	1.00 (ref)	1.00 (ref)	1.00 (ref)
Ideal	92,542	5,684/1,060,192	**0.81 (0.78, 0.84)**	**0.84 (0.81, 0.87)**	**0.87 (0.83, 0.90)**	**0.89 (0.85, 0.92)**
**Healthy diet†**						
Not ideal	109,639	7,364/1,251,566	1.00 (ref)	1.00 (ref)	1.00 (ref)	1.00 (ref)
Ideal	55,854	3,577/640,591	**0.89 (0.86, 0.93)**	**0.95 (0.91, 0.99)**	**0.95 (0.91, 0.99)**	0.99 (0.95, 1.03)
**BMI**						
Not ideal (<18.5 or ≥25, kg/m^2)	92,040	7,675/1,043,619	1.00 (ref)	1.00 (ref)	1.00 (ref)	1.00 (ref)
Ideal (18.5-25, kg/m2)	73,453	3,266/848,538	**0.56 (0.54, 0.58)**	**0.68 (0.65, 0.71)**	**0.72 (0.69, 0.75)**	**0.72 (0.69, 0.75)**

Model 1 adjusted for age and sex;

Model 2 adjusted for Model 1 +ethnicity, education, Townsend deprivation index, household income, baseline mean arterial pressure, alcohol consumption status, and healthy lifestyle categories;

Model 3 adjusted for Model 2 +family history of hypertension, lipid-lowering medication use, diabetes medication use, baseline cardiovascular disease, diabetes, chronic kidney disease, and cancer;

Model 4 adjusted for confounders in Model 3 and included all low-risk lifestyle factors simultaneously and mutually adjusted.

*Categories of physical activity were defined according to the physical activity guidelines for Americans (Jama. 2018;320(19):2020-8).

†Ideal diet was defined as the healthy diet score ≥4; intermediate diet was defined as the healthy diet score =3; poor diet was defined as the healthy diet score <3.

Abbreviations: BMI, body mass index, ref, reference.

Table S7. Joint association of sleep pattern and lifestyle with hypertension incidence among 165,493 participants

	Total No. of participants	No. of cases/ Person-Years	Hazard Ratio (95% CI)	*P* for interaction
**Healthy lifestyle**				0.13
Healthy sleep	30,992	1,245/359,583	1.00 (ref)	
Intermediate sleep	32,488	1,849/373,246	**1.27 (1.18, 1.36)**	
Poor sleep	1,346	108/15,270	**1.66 (1.36, 2.02)**	
**Intermediate lifestyle**				
Healthy sleep	24,418	1,374/280,824	**1.30 (1.20, 1.40)**	
Intermediate sleep	34,527	2,635/392,638	**1.61 (1.50, 1.72)**	
Poor sleep	2,253	241/25,074	**1.98 (1.72, 2.27)**	
**Poor lifestyle**				
Healthy sleep	12,621	941/143,555	**1.59 (1.46, 1.73)**	
Intermediate sleep	24,586	2,250/277,073	**1.82 (1.70, 1.96)**	
Poor sleep	2,262	298/24,894	**2.41 (2.12, 2.74)**	

The models were fully adjusted for age, sex, ethnicity, education, Townsend deprivation index, household income, baseline mean arterial pressure, alcohol consumption, family history of hypertension, baseline cardiovascular disease, diabetes, chronic kidney disease, and cancer.
Abbreviations: ref, reference; CI, confidence interval.

Table S8. Stratified analyses by categories of sleep or lifestyle with incident hypertension among 165,493 participants

Stratified by lifestyle	Poor lifestyle	Intermediate lifestyle	Healthy lifestyle
Poor sleep	1.00 (ref)	1.00 (ref)	1.00 (ref)
Intermediate sleep	**0.75 (0.67, 0.85)**	**0.82 (0.71, 0.93)**	**0.76 (0.63, 0.93)**
Healthy sleep	**0.65 (0.57, 0.74)**	**0.66 (0.57, 0.76)**	**0.61 (0.50, 0.74)**
Stratified by sleep	Poor sleep	Intermediate sleep	Healthy sleep
Poor lifestyle	1.00 (ref)	1.00 (ref)	1.00 (ref)
Intermediate lifestyle	**0.83 (0.70 0.99)**	**0.88 (0.83, 0.93)**	**0.82 (0.75, 0.89)**
Healthy lifestyle	**0.68 (0.54, 0.85)**	**0.70 (0.65, 0.74)**	**0.63 (0.58, 0.69)**

The models were fully adjusted for age, sex, ethnicity, education, Townsend deprivation index, household income, baseline mean arterial pressure, alcohol consumption, family history of hypertension, baseline cardiovascular disease, diabetes, chronic kidney disease, and cancer.
Abbreviations: ref, reference.

Table S9. PAR estimates for hypertension incidence associated with sleep scores and lifestyle scores separately and in combination

	Prevalence (%)	%PAR (95% CI)
Healthy sleep*	41.1	14.7 (12.3, 17.1)
Healthy lifestyle score ≥1	97.4	1.5 (1.0, 1.9)
Healthy lifestyle score ≥2	76.2	7.9 (6.6, 9.2)
Healthy lifestyle score ≥3	39.2	20.1 (17.6, 22.6)
Healthy lifestyle score ≥4	10.4	27.9 (22.6, 33.0)
Healthy sleep and healthy lifestyle score ≥1	40.5	16.9 (14.4, 19.4)
Healthy sleep and healthy lifestyle score ≥2	33.5	21.5 (18.2, 24.8)
Healthy sleep and healthy lifestyle score ≥3	18.7	31.7 (27.6, 35.6)
Healthy sleep and healthy lifestyle score ≥4	5.4	39.1 (33.5, 44.4)
Healthy lifestyle†	39.2	20.1 (17.6, 22.6)
Healthy sleep score ≥1	99.8	0.3 (0.1, 0.4)
Healthy sleep score ≥2	96.5	1.7 (1.2, 2.3)
Healthy sleep score ≥3	78.8	5.9 (4.7, 7.0)
Healthy sleep score ≥4	41.1	14.7 (12.3, 17.1)
Healthy sleep score ≥5	8.5	24.3 (18.3, 30.2)
Healthy lifestyle and healthy sleep score ≥1	39.1	21.4 (18.3, 24.4)
Healthy lifestyle and healthy sleep score ≥2	38.4	22.1 (19.0, 25.2)
Healthy lifestyle and healthy sleep score ≥3	33.1	24.7 (21.4, 28.0)
Healthy lifestyle and healthy sleep score ≥4	18.7	31.7 (27.6, 35.6)
Healthy lifestyle and healthy sleep score ≥5	4.2	40.0 (32.1, 47.4)

PARs and 95% CIs were calculated adjusting for age, sex, ethnicity, education, Townsend deprivation index, household income, baseline mean arterial pressure, alcohol consumption status, family history of hypertension, baseline cardiovascular disease, diabetes, chronic kidney disease, and cancer.

*Healthy sleep defined as healthy sleep score ≥4

†Healthy lifestyle defined as healthy lifestyle score ≥3

Abbreviations: PAR, population-attributable risk; CI, confidence interval.

Table S10. PAR estimates for hypertension incidence associated with sleep scores and lifestyle scores components

	%PAR (95%CI)
All five sleep pattern factors	26.9 (21.1, 32.6)
early chronotype	0.2 (-0.4, 0.9)
ideal sleep duration	6.2 (4.8, 7.7)
never/rarely insomnia	16.6 (13.4, 19.7)
no self-reported snoring	5.5 (3.9, 7.0)
no excessive daytime sleep	1.1 (0.7, 1.5)
All four lifestyle factors	30.9 (25.3, 36.2)
no current smoking	3.9 (3.0, 4.8)
regular physical activity	5.8 (4.0, 7.5)
healthy diet	0.5 (-1.1, 2.0)
not overweight	23.4 (20.9, 25.9)
Four lifestyle and five sleep pattern factors	48.0 (39.7, 55.6)

PARs and 95% CIs were calculated adjusting for age, sex, ethnicity, education, Townsend deprivation index, household income, baseline mean arterial pressure, alcohol consumption status, family history of hypertension, baseline cardiovascular disease, diabetes, chronic kidney disease, and cancer.

Table S11. Association of weighted healthy sleep score with incident hypertension among 165,493 participants

Weighted healthy sleep score	Total No. of participants	No. of cases/ person-years	Model 1	Model 2	Model 3
0~ <2	13,974	1,466/156,274	1.00 (ref)	1.00 (ref)	1.00 (ref)
2~ <3	53,334	4,143/605,505	**0.75 (0.71, 0.80)**	**0.82 (0.77, 0.87)**	**0.85 (0.80, 0.90)**
3~ <4	65,632	3,792/754,101	**0.60 (0.56, 0.64)**	**0.70 (0.66, 0.75)**	**0.74 (0.69, 0.78)**
4~ <5	18,565	934/214,229	**0.53 (0.48, 0.57)**	**0.61 (0.56, 0.66)**	**0.64 (0.59, 0.70)**
5	13,988	606/162,048	**0.48 (0.43, 0.52)**	**0.58 (0.53, 0.64)**	**0.62 (0.56, 0.68)**
P for trend			**<.0001**	**<.0001**	**<.0001**
Per score increment			**0.79 (0.77, 0.80)**	**0.84 (0.82, 0.86)**	**0.86 (0.84, 0.87)**
Quintile of weighted healthy sleep score	Total No. of participants	No. of cases/ person-years	Model 1	Model 2	Model 3
1	34,586	3,056/390,316	1.00 (ref)	1.00 (ref)	1.00 (ref)
2	32,722	2,553/371,464	**0.83 (0.79, 0.88)**	**0.88 (0.84, 0.93)**	**0.89 (0.85, 0.94)**
3	26,009	1,518/298,607	**0.72 (0.68, 0.77)**	**0.80 (0.75, 0.85)**	**0.82 (0.77, 0.88)**
4	39,623	2,274/455,493	**0.66 (0.63, 0.70)**	**0.75 (0.71, 0.80)**	**0.78 (0.74, 0.82)**
5	32,553	1,540/376,277	**0.58 (0.54, 0.61)**	**0.65 (0.61, 0.70)**	**0.68 (0.64, 0.72)**
P for trend			**<.0001**	**<.0001**	**<.0001**
Per score increment			**0.79 (0.77, 0.80)**	**0.84 (0.82, 0.86)**	**0.86 (0.84, 0.87)**

We constructed a weighted sleep score based on the 5 sleep factors by using the equation: weighted sleep score= (β1×factor1 +β2 ×factor 2 +…+β5×factor 5) × (5/sum of the β coefficients).

Model 1 adjusted for age and sex;

Model 2 adjusted for Model 1 + ethnicity, education, Townsend deprivation index, household income, baseline mean arterial pressure, alcohol consumption status, and healthy lifestyle categories;

Model 3 adjusted for Model 2 + family history of hypertension, baseline cardiovascular disease, diabetes, chronic kidney disease, and cancer.
Abbreviations: ref, reference.

Table S12. Joint association of weighted sleep pattern and lifestyle with incident hypertension among 165,493 participants

	Total No. of participants	No. of cases/ Person-Years	Hazard Ratio (95% CI)	*P* for interaction
**Healthy lifestyle**				0.87
Healthy sleep	14,153	504/164,545	1.00 (ref)	
Intermediate sleep	46,885	2,415/540,445	**1.30 (1.18, 1.43)**	
Poor sleep	3,788	283/43,110	**1.58 (1.37, 1.83)**	
**Intermediate lifestyle**				
Healthy sleep	11,927	601/137,622	**1.31 (1.16, 1.48)**	
Intermediate sleep	43,835	3,084/500,122	**1.67 (1.52, 1.83)**	
Poor sleep	5,436	565/60,792	**2.03 (1.80, 2.29)**	
**Poor lifestyle**				
Healthy sleep	6,473	435/74,110	**1.61 (1.42, 1.83)**	
Intermediate sleep	28,246	2,436/319,039	**1.90 (1.73, 2.10)**	
Poor sleep	4,750	618/52,373	**2.55 (2.27, 2.87)**	

We constructed a weighted sleep score based on the 5 sleep factors by using the equation: weighted sleep score= ($\beta1\times$factor1 +$\beta2$ $\times$factor 2 +…+$\beta5\times$factor 5) $\times$ (5/sum of the β coefficients). This weighted score also ranges from 0 to 5 points but considers magnitudes of the adjusted relative risk for each factor in each sleep pattern as a combination of 5 factors.

The models were fully adjusted for age, sex, ethnicity, education, Townsend deprivation index, household income, baseline mean arterial pressure, alcohol consumption, family history of hypertension, baseline cardiovascular disease, diabetes, chronic kidney disease, and cancer.

Abbreviations: ref, reference; CI, confidence interval.

Table S13. Sensitive analysis (N=165,493) for the association of healthy sleep score and healthy lifestyle score with incident hypertension

	Healthy sleep score					*P* for trend	Per score increment
	0-1	2	3	4	5		
Total No. of participants	5,861	29,280	62,321	54,043	13,988		
No. of hypertension cases/person-years	647/65,239	2,416/331,474	4,318/711,482	2,954/621,914	606/162,048		
Main analysis	1.00 (ref)	**0.83 (0.76, 0.91)**	**0.76 (0.69, 0.82)**	**0.65 (0.60, 0.71)**	**0.58 (0.52, 0.65)**	**<.0001**	**0.88 (0.86, 0.90)**
Sensitivity analysis 1	1.00 (ref)	**0.83 (0.76, 0.91)**	**0.76 (0.70, 0.83)**	**0.66 (0.60, 0.72)**	**0.59 (0.52, 0.66)**	**<.0001**	**0.88 (0.86, 0.90)**
Sensitivity analysis 2	1.00 (ref)	**0.90 (0.82, 0.98)**	**0.84 (0.77, 0.92)**	**0.75 (0.68, 0.82)**	**0.67 (0.60, 0.75)**	**<.0001**	**0.91 (0.89, 0.93)**
	Healthy lifestyle score*						
	0	1	2	3	4		
Total No. of participants	4,253	35,216	61,198	47,703	17,123		
No. of hypertension cases/person-years	480/47,198	3,009/398,324	4,250/698,536	2,479/549,542	723/198,557		
Main analysis	1.00 (ref)	**0.77 (0.70, 0.85)**	**0.68 (0.62, 0.75)**	**0.55 (0.50, 0.61)**	**0.48 (0.43, 0.54)**	**<.0001**	**0.84 (0.82, 0.86)**
Sensitivity analysis 1	1.00 (ref)	**0.78 (0.70, 0.85)**	**0.69 (0.63, 0.76)**	**0.56 (0.50, 0.62)**	**0.49 (0.43, 0.55)**	**<.0001**	**0.85 (0.83, 0.86)**
Sensitivity analysis 2	1.00 (ref)	**0.78 (0.70, 0.85)**	**0.70 (0.63, 0.77)**	**0.57 (0.52, 0.63)**	**0.51 (0.45, 0.57)**	**<.0001**	**0.86 (0.84, 0.87)**

Main analysis adjusted for age, sex, ethnicity, education, Townsend deprivation index, household income, baseline mean arterial pressure, alcohol consumption, healthy lifestyle categories, family history of hypertension, baseline diabetes, chronic kidney disease, self-reported physician-diagnosed cardiovascular disease, and cancer;

Sensitivity analysis 1 adjusted for Main analysis + lipid-lowering medication use and diabetes medication use;

Sensitivity analysis 2 adjusted for Sensitivity analysis 1 + baseline OSA risk.

* Adjusted for healthy sleep score categories in Main analysis, Sensitivity analysis 1, and Sensitivity analysis 2.

Abbreviations: ref, reference.

Table S14. Sensitive analysis (N=164,192) for the association of healthy sleep score and healthy lifestyle score with incident hypertension after excluding individuals with hypertension events in the first 2 years of follow-up

	Healthy sleep score					*P* for trend	Per score increment
	0-1	2	3	4	5		
Total No. of participants	5,771	28,968	61,833	53,697	13,923		
No. of hypertension cases/person-years	589/65,135	2,216/331,098	4,015/710,927	2,749/621,507	570/161,979		
Model 1	1.00 (ref)	**0.73 (0.67, 0.80)**	**0.62 (0.57, 0.68)**	**0.50 (0.46, 0.55)**	**0.44 (0.39, 0.49)**	**<.0001**	**0.82 (0.80, 0.84)**
Model 2	1.00 (ref)	**0.80 (0.73, 0.87)**	**0.72 (0.66, 0.79)**	**0.61 (0.56, 0.67)**	**0.55 (0.49, 0.61)**	**<.0001**	**0.87 (0.85, 0.89)**
Model 3	1.00 (ref)	**0.83 (0.76, 0.91)**	**0.76 (0.70, 0.83)**	**0.66 (0.60, 0.72)**	**0.59 (0.52, 0.66)**	**<.0001**	**0.88 (0.86, 0.90)**
	Healthy lifestyle score*						
	0	1	2	3	4		
Total No. of participants	4,191	34,824	60,710	47,430	17,037		
No. of hypertension cases/person-years	444/47,128	2,752/397,881	3,946/697,961	2,318/549,224	679/198,451		
Model 1	1.00 (ref)	**0.68 (0.61, 0.75)**	**0.55 (0.50, 0.60)**	**0.40 (0.36, 0.44)**	**0.32 (0.28, 0.36)**	**<.0001**	**0.76 (0.75, 0.78)**
Model 2	1.00 (ref)	**0.76 (0.68, 0.84)**	**0.66 (0.60, 0.73)**	**0.53 (0.47, 0.58)**	**0.45 (0.40, 0.51)**	**<.0001**	**0.83 (0.81, 0.85)**
Model 3	1.00 (ref)	**0.76 (0.69, 0.84)**	**0.68 (0.62, 0.75)**	**0.55 (0.50, 0.61)**	**0.48 (0.42, 0.54)**	**<.0001**	**0.84 (0.83, 0.86)**

Model 1 adjusted for age and sex;

Model 2 adjusted for Model 1 + ethnicity, education, Townsend deprivation index, household income, baseline mean arterial pressure, alcohol consumption status, and healthy lifestyle categories;

Model 3 adjusted for Model 2 + family history of hypertension, baseline cardiovascular disease, diabetes, chronic kidney disease, and cancer.

* Adjusted for healthy sleep score category in Model 2 and Model 3.

Abbreviations: ref, reference.

Table S15. Sensitive analysis (N=161,044) for the association of healthy sleep score and healthy lifestyle score with incident hypertension after excluding individuals with cardiovascular diseases at baseline

	Healthy sleep score					*P* for trend	Per score increment
	0-1	2	3	4	5		
Total No. of participants	5,619	28,324	60,556	52,823	13,722		
No. of hypertension cases/person-years	568/62,903	2,114/322,279	3,816/694,168	2,644/609,723	540/159,366		
Model 1	1.00 (ref)	**0.72 (0.66, 0.79)**	**0.61 (0.56, 0.66)**	**0.49 (0.45, 0.54)**	**0.42 (0.37, 0.47)**	**<.0001**	**0.82 (0.80, 0.83)**
Model 2	1.00 (ref)	**0.78 (0.71, 0.86)**	**0.70 (0.64, 0.77)**	**0.60 (0.55, 0.66)**	**0.52 (0.46, 0.59)**	**<.0001**	**0.86 (0.84, 0.88)**
Model 3	1.00 (ref)	**0.79 (0.72, 0.87)**	**0.72 (0.66, 0.78)**	**0.62 (0.56, 0.67)**	**0.54 (0.48, 0.61)**	**<.0001**	**0.87 (0.85, 0.89)**
	Healthy lifestyle score*						
	0	1	2	3	4		
Total No. of participants	4,051	34,039	59,545	46,618	16,791		
No. of hypertension cases/person-years	404/45,446	2,631/387,208	3,762/682,275	2,227/538,520	658/194,990		
Model 1	1.00 (ref)	**0.71 (0.64, 0.79)**	**0.57 (0.51, 0.63)**	**0.42 (0.37, 0.46)**	**0.33 (0.29, 0.37)**	**<.0001**	**0.76 (0.75, 0.78)**
Model 2	1.00 (ref)	**0.78 (0.70, 0.87)**	**0.68 (0.61, 0.75)**	**0.55 (0.49, 0.61)**	**0.48 (0.42, 0.54)**	**<.0001**	**0.84 (0.82, 0.85)**
Model 3	1.00 (ref)	**0.78 (0.70, 0.86)**	**0.68 (0.62, 0.76)**	**0.55 (0.50, 0.62)**	**0.48 (0.43, 0.55)**	**<.0001**	**0.84 (0.83, 0.86)**

Model 1 adjusted for age and sex;

Model 2 adjusted for Model 1+ ethnicity, education, Townsend deprivation index, household income, mean arterial pressure, alcohol consumption, and healthy lifestyle categories;

Model 3 adjusted for Model 2+ family history of hypertension, baseline diabetes, chronic kidney disease, and cancer.

* Adjusted for healthy sleep score category in Model 2 and Model 3.

Abbreviations: ref, reference.

Table S16. Joint association of sleep pattern and lifestyle with incident hypertension after excluding individuals with cardiovascular diseases at baseline (N=161,044)

	Total No. of participants	No. of cases/ Person-Years	Hazard Ratio (95% CI)	*P* for interaction
**Healthy lifestyle**				0.28
Healthy sleep	30,415	1,133/353,570	1.00 (ref)	
Intermediate sleep	31,689	1,654/365,092	**1.27 (1.17, 1.37)**	
Poor sleep	1,305	98/14,849	**1.69 (1.37, 2.07)**	
**Intermediate lifestyle**				
Healthy sleep	23,884	1,226/275,526	**1.29 (1.19, 1.40)**	
Intermediate sleep	33,507	2,328/382,627	**1.62 (1.50, 1.74)**	
Poor sleep	2,154	208/24,122	**2.05 (1.77, 2.38)**	
**Poor lifestyle**				
Healthy sleep	12,246	825/139,994	**1.59 (1.45, 1.74)**	
Intermediate sleep	23,684	1,948/268,728	**1.83 (1.70, 1.97)**	
Poor sleep	2,160	262/23,932	**2.62 (2.29, 3.01)**	

The models were fully adjusted for age, sex, ethnicity, education, Townsend deprivation index, household income, baseline mean arterial pressure, alcohol consumption, family history of hypertension, baseline diabetes, chronic kidney disease, and cancer.
Abbreviations: ref, reference; CI, confidence interval.

Table S17. Joint association of sleep pattern and lifestyle with incident hypertension after excluding individuals with hypertension events in the first 2 years of follow-up (N=164,192)

	Total No. of participants	No. of cases/ Person-Years	Hazard Ratio (95% CI)	*P* for interaction
**Healthy lifestyle**				0.18
Healthy sleep	30,861	1,175/359,429	1.00 (ref)	
Intermediate sleep	32,275	1,724/372,994	**1.25 (1.16, 1.35)**	
Poor sleep	1,331	98/15,252	**1.59 (1.30, 1.96)**	
**Intermediate lifestyle**				
Healthy sleep	24,260	1,278/280,636	**1.29 (1.19, 1.39)**	
Intermediate sleep	34,225	2,443/392,283	**1.60 (1.49, 1.71)**	
Poor sleep	2,225	225/25,042	**2.01 (1.74, 2.31)**	
**Poor lifestyle**				
Healthy sleep	12,499	866/143,421	**1.58 (1.44, 1.72)**	
Intermediate sleep	24,301	2,064/276,748	**1.80 (1.68, 1.94)**	
Poor sleep	2,215	266/24,841	**2.35 (2.06, 2.69)**	

The models were fully adjusted for age, sex, ethnicity, education, Townsend deprivation index, household income, baseline mean arterial pressure, alcohol consumption, family history of hypertension, baseline cardiovascular disease, diabetes, chronic kidney disease, and cancer. Abbreviations: ref, reference; CI, confidence interval.

Table S18. Cohort characteristics for participants with full or missing data on sleep pattern

Characteristics	Full data on sleep pattern	Missing data on sleep pattern
Number of participants	165,493	39,291
Age, mean (SD), y	53.6 (8.0)	54.1 (8.1)
Male, n (%)	62,302 (37.6)	16,086 (40.9)
Deprivation fifth, n (%)		
First (least deprived)	34,220 (20.7%)	6,640 (16.9%)
Second	34,034 (20.6%)	6,914 (17.6%)
Third	33,543 (20.3%)	7,365 (18.7%)
Forth	32,744 (19.8%)	8,162 (20.8%)
Fifth (most deprived)	30,749 (18.6%)	10,155 (25.9%)
Missing	203 (0.1%)	55 (0.1%)
BMI, mean (SD), kg/m2	26.0 (4.1)	26.1 (4.3)
Education, n (%)		
College or university	65,178 (39.4%)	13,287 (33.8%)
Vocational	16,446 (9.9%)	3,707 (9.4%)
Upper secondary	20,770 (12.6%)	4,307 (11.0%)
Lower secondary	45,214 (27.3%)	9,764 (24.9%)
Others	17,009 (10.3%)	5,108 (13.0%)
Unknown	876 (0.5%)	3,118 (7.9%)
Ethnicity, n (%)		
White	157,142 (95.0%)	35,519 (90.4%)
Mixed	1,144 (0.7%)	337 (0.9%)
Asian	2,855 (1.7%)	987 (2.5%)
Black	1,932 (1.2%)	798 (2.0%)
Chinese	601 (0.4%)	194 (0.5%)
Others	1,418 (0.9%)	574 (1.5%)
Missing	401 (0.2%)	882 (2.2%)
Household income, £, n (%)		
<18,000	23,083 (14.0%)	7,956 (20.3%)
18,000-30,999	32,861 (19.9%)	7,600 (19.3%)
31,000-51,999	42,044 (25.4%)	7,610 (19.4%)
52,000-100,000	38,518 (23.3%)	5,569 (14.2%)
>100,000	11,353 (6.9%)	1,473 (3.8%)
Missing	17,634 (10.7%)	9,083 (23.1%)
Moderate alcohol consumption, n (%)	91,452 (55.3)	19,987 (50.9)
Systolic blood pressure, mean (SD), mm Hg	123.8 (9.8)	123.8 (9.9)

Diastolic blood pressure, mean (SD), mm Hg	76.2 (7.0)	76.1 (7.1)
Cardiovascular disease, n (%)	4,449 (2.7)	1,368 (3.5)
Diabetes mellitus, n (%)	3,764 (2.3)	1,153 (2.9)
Chronic kidney disease, n (%)	1,428 (0.9)	441 (1.1)
Cancer, n (%)	13,766 (8.3)	3,227 (8.2)
Hypertension cases, n (%)	10941 (6.6)	3353 (8.5)

Abbreviations: SD, standard deviation; BMI, body mass index.

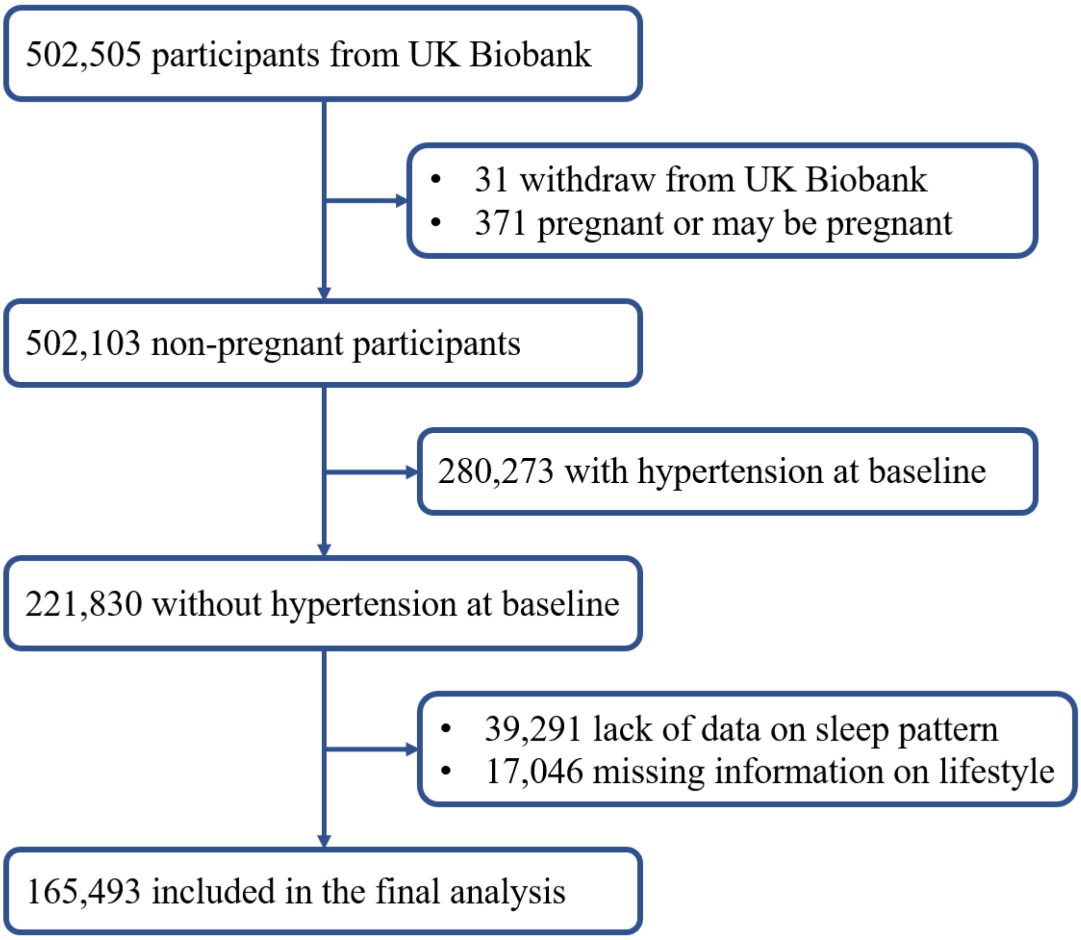

Figure S1. Flowchart of participants enrolment.

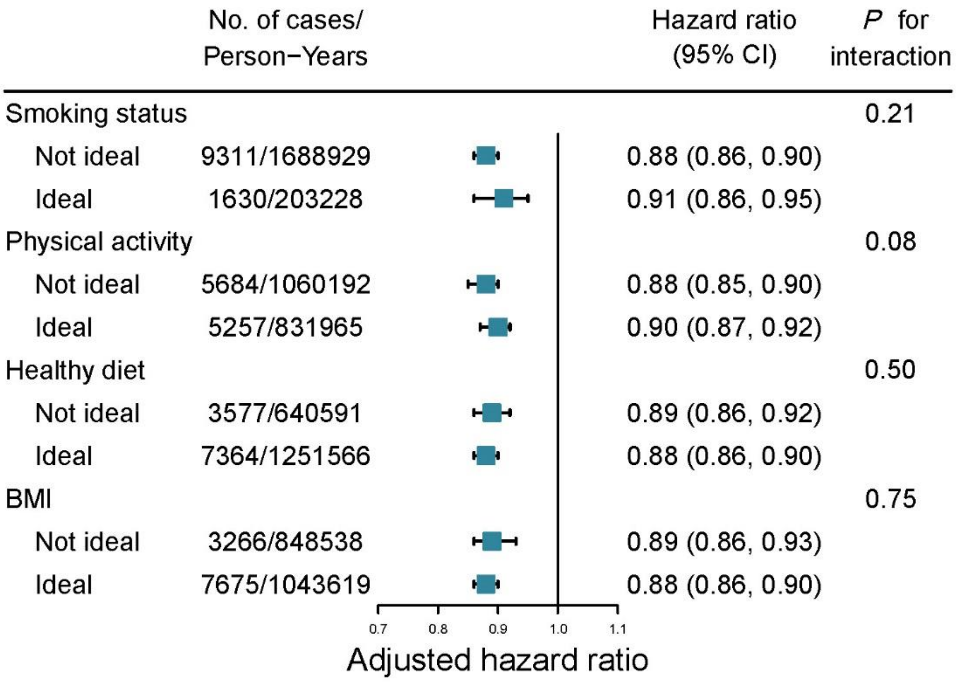

Figure S2. Associations of per healthy sleep score increment with incident hypertension stratified by lifestyle components among 165,493 participants.

Abbreviations: BMI, body mass index; HR, hazard ratio; CI, confidence interval.
